# Association of smoking cessation with dynapenia among older lifetime smokers in Korea

**DOI:** 10.18332/tid/191822

**Published:** 2024-08-31

**Authors:** Keunjoong Yoo, Yong Soon Park, Hye Jin Kim, Jeong Hyeon Kim

**Affiliations:** 1 Department of Family Medicine, Chuncheon Sacred Heart Hospital, Hallym University College of Medicine, Chuncheon, Republic of Korea; 2 Institute of New Frontier Research, Hallym University College of Medicine, Chuncheon, Republic of Korea

**Keywords:** dynapenia, smoking, older adults, smoking cessation, Korea

## Abstract

**INTRODUCTION:**

Muscle strength is known to play an important role in the health of older adults. The health burden of cigarette smoking among older adults remains significant. We investigated the association between smoking cessation and dynapenia among older lifetime smokers in Korea.

**METHODS:**

This study is a secondary dataset analysis of cross-sectional data from the

Korea National Health and Nutrition Examination Survey (KNHANES) 2016– 2019. We included 1450 participants aged 65–79 years, excluding those who had never smoked. Dynapenia was defined as grip strength <28 kg for men and <18 kg for women based on the Asian Working Group for Sarcopenia 2019 criteria. Multivariable logistic regression analysis evaluated the association between smoking cessation and dynapenia.

**RESULTS:**

Compared with current smokers, the adjusted odds ratio (AOR) of dynapenia in former smokers was 0.66 (95% CI: 0.44–0.99). The AORs for smoking cessation periods of ≤10 years, 10–20 years, 20–30 years, and >30 years were 0.67 (95% CI: 0.39–1.16), 0.61 (95% CI: 0.36–1.03), 0.65 (95% CI: 0.37–1.14), and 0.52 (95% CI: 0.25–1.06), respectively. The AOR for dynapenia significantly decreased with the years since smoking cessation (p for trend=0.043).

**CONCLUSIONS:**

Our findings suggest that smoking cessation can reduce the likelihood of dynapenia among older lifetime smokers, with a decreasing likelihood trend associated with longer cessation periods.

## INTRODUCTION

Low muscle strength, or dynapenia, is strongly associated with diverse adverse health outcomes^1^ and is a well-known clinical characteristic of sarcopenia^2-4^. Current diagnostic criteria for diagnosing sarcopenia in Asia^2^, Europe^3^, and the United States^4^ include an assessment of muscle strength, mass, and physical performance. Given the simplicity and low assessment cost, grip strength is widely used to measure muscle strength. Decreased grip strength is associated with increased mortality, disability, and morbidity rates. A prospective cohort study of community-dwelling adults aged 35–70 years from 17 countries showed that grip strength was inversely associated with all-cause mortality, cardiovascular mortality, myocardial infarction, and stroke^5^. A prospective cohort study of JapaneseAmerican men, with a 25-year follow-up, showed that poor grip strength during midlife was highly predictive of functional limitation and disability in older age^6^. Another prospective cohort study of men in the USA that involved a 25-year follow-up, showed that independent of muscle mass and physical activity, lower and declining grip strengths were associated with an increased mortality risk^7^.

Cigarette smoking is a modifiable risk factor that increases the risk for various types of malignancies, such as lung, laryngeal, and esophageal cancer, stroke, cardiovascular diseases, and chronic obstructive pulmonary disease^8^. Worldwide, the prevalence of smoking has decreased significantly over the past few decades and is possibly attributable to anti-smoking campaigns. Nevertheless, in 2019, the number of current smokers worldwide peaked at 1.1 billion, and smoking led to 7.69 million deaths and 200 million disability-adjusted life years^9^. Smoking-related illnesses constitute a global health concern, especially in the aging population, even though smoking cessation can reverse the harmful effects of smoking^10^. Moreover, although the association of smoking status with dynapenia has been investigated, the results have been inconsistent. Some studies reported that smoking is associated with dynapenia^11,12^, whereas other studies revealed no association^13^. Furthermore, unlike other smoking-related diseases, there is limited research on the relationship between the duration of smoking cessation and dynapenia.

In this era of increasing population of aged people, health issues among the older population have become increasingly critical areas. In addition to ascertaining the harmful effects of smoking and the benefits of smoking cessation, it is crucial to understand how smoking cessation affects skeletal muscle strength. Thus, in this study, we aimed to investigate the association of smoking cessation with dynapenia and the influence of the duration since smoking cessation on dynapenia among older lifetime smokers in Korea.

## METHODS

### Study population

**Figure 1:**
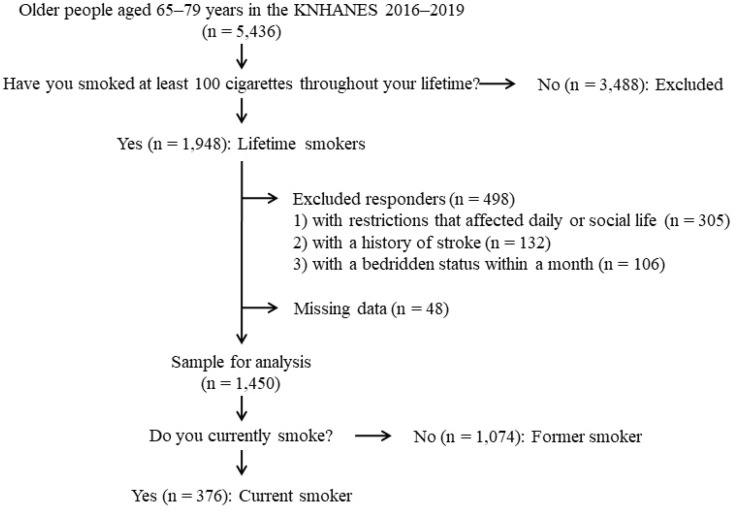
Flowchart depicting selection of the study participants

This secondary dataset analysis was based on the data obtained from the Korea National Health and Nutrition Examination Survey (KNHANES) 2016–2019, which is a nationwide cross-sectional survey conducted by the Korea Disease Control and Prevention Agency (KDCA) since 1998 to assess the health and nutrition status of Korean people. The KNHANES used a multistage stratified cluster sampling method to create a sample representative of the civilian non-institutionalized population in Korea. A total of 32379 participants were enrolled in the KNHANES from 2016 to 2019. We included 5436 older adults aged 65–79 years and excluded never smokers (n=3488) who had smoked fewer than 100 cigarettes in their lifetime. Of the remaining 1948 participants, we excluded those with restrictions that affect daily or social life (n=306), those with a history of stroke (n=132), those with a bedridden status within a month (n=106), and those with missing data about grip strength or smoking status (n=48). Finally, 1450 participants were selected for the analyses. The selection process of the study participants is depicted in Figure 1. The KNHANES was conducted in accordance with Ethical Principles for Medical Research Involving Human Subjects. All participants provided written informed consent prior to participating in the KNHANES, the data from which were de-identified before being made publicly available. This study was approved by the Institutional Review Board of Hallym University Chuncheon Sacred Heart Hospital (CHUNCHEON 2022-07-007-001).

### Data collection and measurement

*Grip strength and dynapenia*

Grip strength was measured using a digital grip strength dynamometer (TKK 5401; Takei Scientific Instruments Co., Ltd., Tokyo, Japan). Well-trained examiners sequentially measured the grip strength of both hands, with six measurements obtained in triplicate per hand. Participants were instructed to grip the dynamometer as firmly as possible for 3 seconds in a standing position with their arms hanging down at their sides. A 60-second resting interval was allowed between measurements. The highest grip strength of both hands was used for the analysis. According to the 2019 consensus update on sarcopenia diagnosis and treatment written by the Asian Working Group for Sarcopenia,^2^ we defined dynapenia as a grip strength of <28 kg for men and <18 kg for women.

*Smoking status*

Lifetime cigarette smokers comprised current and former smokers. Individuals who had smoked at least 100 cigarettes in their lifetime and who currently smoked were classified as current smokers, whereas those who had smoked at least 100 cigarettes in their lifetime but did not currently smoke were classified as former smokers. For further analysis, former smokers were divided into four groups stratified by 10-year intervals for the duration (number of years) since smoking cessation.

*Covariables*

We selected factors known to be associated with dynapenia or sarcopenia from previous studies, focusing them on demographic and socioeconomic, lifestyle, and health status variables^2,14-17^.

Demographic and socioeconomic variables included age, sex, education level, employment status, household income, marital status, and living status. Age was classified into three categories based on 5-year intervals. The education level was classified as high school or higher and middle school or lower. Based on their employment status, the participants were divided into two groups: employed (those who worked for more than 1 hour for income or at least 18 hours a week as unpaid family workers, including temporary leave of absence) and unemployed. Household income was categorized into lowest, middle (2nd, 3rd, and 4th quintiles), and highest quintiles. Unmarried, separated, widowed, and divorced participants were assigned the ‘no spouse’ status. Living status was classified as either living alone or cohabiting. Lifestyle factors included alcohol consumption, aerobic physical activity, and resistance exercise. Alcohol consumption was divided into two categories: at least two days of drinking per week and fewer than two days per week, regardless of the amount of alcohol consumed. Aerobic physical activity was defined as follows: moderate-intensity physical activity for 150 min or more per week, high-intensity physical activity for 75 min or more per week, or a mix of moderate-intensity and high-intensity physical activity (1 min of high intensity is equivalent to 2 min of moderate intensity) per week. Resistance exercise practice was defined as training for at least two days per week and was based on the type of muscle exercise, such as push-ups, sit-ups, dumbbell lifting, and barbell lifting.

Health status was evaluated based on body mass index (BMI), unintentional weight loss, chewing discomfort, stress, and comorbidity. Obesity was defined^18^ as a BMI of at least 25 kg/m^2^. Unintentional weight loss was defined as losing at least 3 kg in the past year without any effort to lose weight. Chewing discomfort was defined as a positive response to the question: ‘Do you feel uncomfortable chewing food owing to problems in your oral cavity, teeth, dentures, and gums?’. To assess perceived stress, participants were asked: ‘How much stress do you usually feel during your daily life?’. Responses were recorded on a 4-point scale: almost none, some, high, and very high. Participants were considered stressed if they reported high or extremely high-stress levels. Comorbidity included cancers of the stomach, liver, colon, breast, cervix, lung, and thyroid, ischemic heart disease, hypertension, diabetes mellitus, osteoarthritis, and depression. Cancer and ischemic heart disease were defined as a history of prior diagnosis by a physician regardless of medication use, and the rest were defined as cases receiving pharmacological treatment following a physician’s diagnosis. For comprehensive details on variable measurement, we referred to the guidelines on the KNHANES website (knhanes.kdca.go.kr/).

### Statistical analysis

Given the complex sampling design of the KNHANES, all statistical analysis methods used complex sample analyses that accounted for weight, stratified variables, and cluster sampling. All estimates were weighted based on the sample rate, response rate, and the age, sex, and regional proportions of the reference population. Categorical variables are presented as unweighted frequencies, weighted proportions (%), and standard errors (SEs). The Rao-Scott chi-squared test was used to compare participants’ general characteristics according to dynapenia. Logistic regression analyses were used to identify the relationship between the prevalence of dynapenia and related factors among older lifetime smokers. A multivariable analysis model was developed with dynapenia as the outcome variable and risk factors with p<0.05 in the univariate analysis, including smoking status as an explanatory variable. In addition, a trend test was conducted by modeling the main independent variables as a continuous variable for smoking cessation periods. All tests were two-sided, and a p<0.05 was considered statistically significant. All statistical analyses were performed using SPSS version 27 (IBM Corp., Armonk, NY, USA).

## RESULTS

The general characteristics of the study participants who were lifetime smokers aged ≥65 years by the presence of dynapenia are shown in Table 1. Of the 1450 study participants, 1370 (94.1%) were males, 376 (26.5%) were current smokers, and 218 (14.8%) were affected by dynapenia. The participants with dynapenia were older, had a higher percentage of females, a lower education level, a lower household income, and a higher proportion without a spouse than those without dynapenia. Moreover, the dynapenia group experienced chewing discomfort, stress, and unintentional weight loss more commonly, performed aerobic physical activity and resistance exercise less frequently, and had more current smokers, a lower BMI, and a higher prevalence of depression than the non-dynapenia group. The percentages of former smokers were 63.4% and 75.2%, respectively, in the dynapenia and non-dynapenia groups.

**Table 1:** General characteristics of participants according to dynapenia in Korean lifetime smokers aged 65–79 years from the Korea National Health and Nutrition Examination Survey 2016–2019 (N=1450)

*Characteristics*	*Categories*	With dynapenia* (N=218)			Without dynapenia* (N=1232)			*p*
**Age** (years)	75−79	92	36.5	3.7	267	19.4	1.2	<0.001
	70−74	88	42.0	3.9	400	30.8	1.5	
	65−69	38	21.5	3.4	565	49.8	1.6	
**Sex**	Female	24	11.8	2.6	56	4.9	0.8	0.003
**Education level**	≤ Middle school	144	73.4	3.8	624	51.6	1.7	<0.001
**Employment**	No	100	53.6	4.3	616	52.1	1.7	0.755
**Household income**	Lowest quintile	106	47.2	3.8	335	25.6	1.3	<0.001
	Middle	104	47.3	3.9	750	62.8	1.6	
	Highest quintile	8	5.5	2.3	140	11.6	1.1	
**Spouse**	No	46	22.4	3.4	165	12.9	1.1	0.002
**Living alone**	Yes	34	13.5	2.7	167	12.0	1.0	0.577
**Smoking status**	Current smoker	79	36.6	3.7	297	24.8	1.4	0.002
	Former smoker	139	63.4	3.7	935	75.2	1.4	
**Alcohol consumption** (days/week)	≥2	71	32.9	3.6	461	37.9	1.7	0.203
**Regular aerobic physical activity**	No	138	72.1	3.6	705	59.0	1.7	0.002
**Resistance exercise** (days/week)	<2	161	78.9	3.8	801	66.7	1.6	0.009
Body mass index (kg/m^2^)	≥25	37	18.3	3.3	462	36.3	1.6	<0.001
**Unintentional weight loss** (kg/year)	≥3	38	16.3	2.9	92	7.3	0.8	<0.001
**Chewing discomfort**	Yes	109	43.6	3.8	426	33.4	1.6	0.010
**Stress**	Yes	39	20.3	3.6	125	10.6	1.0	0.002
**Comorbidity**	Cancer	14	6.2	1.8	129	9.7	0.9	0.123
	Ischemic heart disease	18	7.0	1.8	124	9.8	1.6	0.218
	Hypertension	97	43.3	3.9	598	47.8	1.6	0.297
	Diabetes mellitus	54	23.4	3.2	275	21.4	1.3	0.550
	Osteoarthritis	13	5.3	1.7	56	4.2	0.6	0.528
	Depression	5	2.4	1.2	9	0.7	0.2	0.033

* Data are expressed as: unweighted frequencies; weighted percentages; and standard errors. P-values are those of the Rao-Scott chi-squared test for proportions

A multivariable logistic regression analysis was conducted to examine the association between dynapenia and variables that were determined to be significant (p<0.05) in the univariate analysis (Table 2). The analysis was adjusted for age and sex in the first model and for variables that were significant in the univariate analysis in the second model. Former smokers were less likely to have dynapenia compared to current smokers in the age- and sexadjusted model (AOR=0.55; 95% CI: 0.38–0.78). After full adjustment, the association was attenuated but remained significant (AOR=0.66; 95% CI: 0.44– 0.99). Besides smoking status, participants with older age (AOR=3.60; 95% CI: 2.09–6.18, for 75–79 years, and AOR=3.14; 95% CI: 1.88–5.24, for 70–74 years), lower education level (AOR=1.52; 95% CI: 1.00–2.31), and less frequent aerobic physical activity (AOR=1.57; 95% CI: 1.04–2.39) were more likely to have dynapenia than the reference group in the fully adjusted analysis. Participants with higher BMIs were less likely to have dynapenia than those with lower BMI (AOR=0.40; 95% CI: 0.24–0.66).

**Table 2:** Multivariable logistic regression analysis of factors associated with dynapenia in Korean lifetime smokers aged 65–79 years from the Korea National Health and Nutrition Examination Survey 2016–2019 (N=1450)

*Variables*	*Categories*	*Model 1*			*Model 2*		
		*AOR*	*95% CI*	*p*	*AOR*	*95% CI*	*p*
**Age** (years)	75−79	4.41	2.78−6.98	<0.001	3.60	2.09−6.18	<0.001
	70−74	3.11	1.97−4.91	<0.001	3.14	1.88−5.24	<0.001
	65−69 ®	1			1		
**Sex**	Female	2.58	1.40−4.77	0.002	1.42	0.64−3.15	0.379
	Male ®	1			1		
**Education level**	≤ Middle school	2.24	1.47−3.40	<0.001	1.52	1.00−2.31	0.047
	≥ High school ®	1			1		
**Household income**	Lowest quintile	2.72	1.07−6.89	0.035	1.73	0.66−4.52	0.258
	Middle	1.39	0.55−3.51	0.485	1.05	0.40−2.77	0.909
	Highest quintile ®	1			1		
**Spouse**	No	1.56	0.94−2.58	0.081	1.13	0.65−1.98	0.649
	Yes ®	1			1		
**Smoking status**	Former smoker	0.55	0.38−0.78	0.001	0.66	0.44−0.99	0.047
	Current smoker ®	1			1		
**Aerobic physical activity**	None	1.62	1.10−2.38	0.013	1.57	1.04−2.39	0.032
	Regular ®	1			1		
**Resistance exercise** (days/week)	<2	1.70	1.05−2.76	0.031	1.32	0.75−2.31	0.327
	≥2 ®	1			1		
**Body mass index** (kg/m^2^)	≥25	0.39	0.24−0.62	<0.001	0.40	0.24−0.66	<0.001
	<25 ®	1			1		
**Unintentional weight loss** (kg/year)	≥3	2.35	1.42−3.92	<0.001	1.52	0.91−2.54	0.104
	<3 ®	1			1		
**Chewing discomfort**	Yes	1.56	1.11−2.19	0.009	1.40	0.97−2.02	0.065
	No ®	1			1		
**Stress**	Yes	2.38	1.41−4.02	0.001	1.75	0.92−3.34	0.086
	No ®	1			1		
**Depression**	Yes	3.12	0.74−13.14	0.120	2.71	0.50−14.78	0.246
	No ®	1			1		

AOR: adjusted odds ratio. Model 1: adjusted for age and sex. Model 2: adjusted for variables that were significant in the univariate analysis. Reference categories.

**Figure 2:**
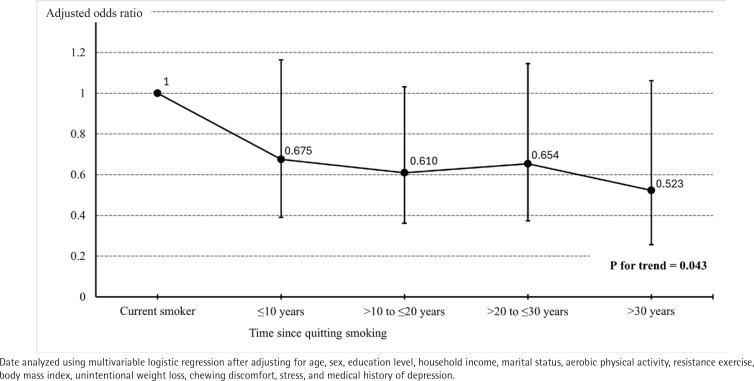
Effects of smoking cessation on dynapenia according to the duration of abstinence among older lifetime smokers (round dots, adjusted odds ratios; upper and lower bars, 95% CIs) from the Korea National Health and Nutrition Examination Survey 2016–2019 (N=1450)

The logistic regression analysis revealed that the AOR for dynapenia decreased with longer durations of smoking cessation (Figure 2). Compared to current smokers, each additional decade of smoking cessation was associated with a statistically significant decrease in the AOR for dynapenia (p for trend=0.043), with an estimated likelihood reduction of approximately 14% per decade (AOR=0.86; 95% CI: 0.74–0.99).

## DISCUSSION

This study aimed to evaluate the impact of smoking cessation on dynapenia among older Korean lifetime smokers, utilizing data from the KNHANES conducted between 2016 and 2019. A multivariable logistic regression analysis identified factors significantly associated with a higher likelihood of reporting dynapenia, including age, education level, obesity, levels of aerobic physical activity, and smoking status. The analysis indicated that former smokers had a 33.5% lower likelihood of dynapenia compared to current smokers. Moreover, the likelihood of dynapenia decreased by approximately 14.2% for every additional ten years of smoking cessation. These findings suggest that smoking cessation significantly reduces the likelihood of dynapenia among older lifetime smokers in Korea, with longer durations of cessation being associated with a greater reduction in likelihood.

Several studies have explored the association between smoking status and dynapenia, yielding inconsistent results. An observational cohort study of Korean men aged ≥65 years found that current smokers had a higher risk of grip strength below 31.4 kg compared to non-smokers^11^. In a cross-sectional study of Japanese men aged 20-79 years, cigarette smoking was associated with grip strength, with heavy smokers exhibiting lower grip strength than light smokers^12^. Conversely, an observational cohort study of older adults in the USA found no association between grip strength and smoking status^13^. These studies compared the muscle strength of current or former smokers with that of non-smokers, using nonsmokers as the reference group. However, in clinical settings, there is a significant demand for information regarding the benefits that older smokers may gain from smoking cessation. Therefore, we excluded nonsmokers from our study and focused on investigating the effects of smoking cessation on dynapenia among lifetime smokers.

Understanding the benefits of smoking cessation facilitates the promotion of tobacco control policies. Several studies have investigated the timeline of various health risks associated with smoking cessation. A cohort study of male British doctors reported that smoking cessation at age 60, 50, 40, or 30 years gained approximately 3, 6, 9, or 10 years of life expectancy, respectively^19^. Peto et al.^20^ demonstrated that risk ratios for lung cancer among former smokers, compared to current smokers, were 0.66, 0.44, 0.20, and 0.10 for those with <10, 10–19, 20–29, and ≥30 years of smoking cessation, respectively^20^. A retrospective analysis of the Framingham Heart Study data revealed that the hazard ratio for cardiovascular diseases among former smokers who quit within the first five years compared with current smokers was 0.61 (95% CI: 0.49–0.76), with a gradual decline after that^21^. Unlike other smoking-related diseases, research on the impact of smoking cessation on muscle strength, based on the duration since cessation, is limited. This study is significant as it suggests that the AOR for dynapenia tends to decrease with the increasing duration of smoking cessation.

Some studies have investigated the effects of smoking cessation on body weight and composition. A nationwide observational study of USA adults demonstrated that individuals who quit smoking gained significantly more weight over ten years compared to those who continued smoking, with men gaining an average of 4.4 kg and women gaining 5.0 kg^22^. A one-year longitudinal study of Israeli adult smokers reported significant increases in body weight (4.43 kg), fat mass (3.15 kg), appendicular skeletal muscle mass index (0.27 kg/m^2^), and grip strength (3.6 kg) among those who quit smoking compared to those who continued^23^. Additionally, a 16-month longitudinal study of postmenopausal women indicated that smoking cessation might be associated with weight gain and increases in fat and muscle mass^24^. Although weight gain from smoking cessation may pose health risks, such as potential metabolic complications, it can also lead to increased muscle mass, contributing to improved overall strength and muscle function. Further prospective clinical studies are needed to investigate the potential for muscle strength improvement following smoking cessation.

Several studies have investigated the mechanisms by which smoking affects muscle quantity and quality. Smoking-induced free radicals cause oxidative damage to skeletal muscle^25,26^ and impair muscle protein synthesis^26^, which can result in the loss of muscle mass and muscle dysfunction^27-29^. Consequently, smoking cessation could increase muscle mass and strength by mitigating these detrimental mechanisms. By quitting smoking, the production of free radicals decreases, thereby reducing oxidative damage to muscle tissues. This reduction in oxidative stress can lead to the recovery of muscle health and the prevention of further muscle degradation. Smoking cessation allows the normal processes of muscle protein synthesis to resume, increasing muscle mass and strength. Restoring protein synthesis is crucial for repairing and growing muscle tissue, enhancing overall muscle functionality.

### Strengths and limitations

The present study has some inherent limitations. First, as a cross-sectional study, it is not possible to demonstrate causality. Further prospective studies are required to confirm the causal relationship between smoking cessation and dynapenia. Second, owing to the nature of secondary data analysis using the KNHANES data, some factors that may affect the relationship between muscle strength and smoking cessation might not have been included. Third, the results may be influenced by selection and recall biases, as the KNHANES targets relatively healthy individuals and relies on self-reported questionnaires. Fourth, although older age was associated with smoking cessation, there may have been bias owing to the high mortality and morbidity associated with smoking. Finally, since this study is for Korea, its findings may have limited generalizability to other countries. Despite these limitations, this study also has several strengths. Our findings were based on a large-scale, nationwide dataset that is population-based and representative of community-dwelling older adults in Korea. All analyses in this study were conducted using sample weights and were adjusted for the complex sample design of the survey. Therefore, these results can be generalized to the Korean elderly population. Additionally, this study analyzed the association between smoking cessation and dynapenia by comparing current and former smokers. It is significant being the first investigation to examine the trend of dynapenia changes according to the duration of smoking cessation.

## CONCLUSIONS

We demonstrated that former smokers had a lower likelihood of dynapenia than current smokers among older Korean adults. There was a significant trend indicating that the likelihood of dynapenia decreased as the duration of smoking cessation increased. Primary healthcare providers should pay attention to the potential benefits of smoking cessation for reducing the likelihood of dynapenia, even in older populations. Further prospective studies are needed to clarify the causal relationship between muscle strength and smoking cessation and to illustrate the time course of the likelihood of dynapenia following smoking cessation.

## Data Availability

The KNHANES data supporting this research are available (https://knhanes.kdca.go.kr/knhanes/main.do ).
